# Measuring dispositional empathy in South African children

**DOI:** 10.1017/neu.2024.19

**Published:** 2024-04-25

**Authors:** Susan Malcolm-Smith, Lea-Ann Pileggi, Raphaella Lewis

**Affiliations:** 1 Applied Cognitive Science and Experimental Neuropsychology Team, Department of Psychology, University of Cape Town, Cape Town, South Africa; 2 Neuroscience Institute, University of Cape Town, Cape Town, South Africa

**Keywords:** Parent-report, cognitive empathy, affective empathy, LMIC, psychometrics

## Abstract

**Objective:**

Empathy is a key factor to examine in development, because of its predictive associations with both aggression and successful prosocial behaviour. However, established measures of empathy for Low-to-Middle Income Countries, including South Africa, are lacking. In children, parent-report measures are key. However, a local study examining empathy and aggression (Malcolm-Smith *et al*., 2015) found poor psychometric performance for a widely used parent-report measure of dispositional empathy, the Griffith Empathy Measure (GEM). We thus investigated which of two questionnaires measuring dispositional cognitive and affective empathy perform better in this context.

**Method:**

We contrasted internal consistency reliability of a simplified version of the GEM (SGEM; *n* = 160) and a parent-report version of the Questionnaire of Cognitive and Affective Empathy (QCAE; *n* = 440) in a low-mid socio-economic status sample. Convergence between the measures and factor structure were also assessed.

**Results:**

The parent-report version of the QCAE performed well as a measure of child dispositional cognitive and affective empathy, with good reliability (overall α = 0.90 vs. SGEM α = .63), and confirmatory factor analysis supporting the two-factor structure. The SGEM’s reliability and failure to correlate with QCAE indicated poor psychometric performance.

**Conclusion:**

This is the first psychometric evaluation of the QCAE as a parent-report measure, and our results indicate that it should prove useful for future assessments of dispositional empathy in children across a variety of contexts.

Significant outcomes

- The simplified GEM did not evidence adequate reliability, and the questionnaire and its subscale scores did not converge with the QCAE total or subscale scores, indicating questionable validity.
- The QCAE performed well, with good reliability and CFA confirming the important two-factor structure (cognitive and affective empathy subscales) in this non-WEIRD sample.
- The parent-report version of the QCAE should be a useful measure of child dispositional empathy for LMIC contexts.

Limitations

- The original five-factor structure of the QCAE was not supported – the peripheral responsivity scale (as in other studies) did not emerge.
- The sample was urban and schooled in English. Findings from rural populations and translated versions would be valuable.
- External validation of the QCAE for this context remains to be established.

## Introduction

The examination of empathy in development is important for a number of reasons. Empathic skills facilitate successful social interactions and relationships (Eisenberg & Fabes, 1990; Zahn-Waxler *et al*., 1992; Decety & Cowell, 2014). In contrast, deficits in empathy contribute to significant individual and societal problems. A substantial body of research in High Income Countries (HICs) has demonstrated an important association between reduced levels of empathy and higher levels of aggressive and antisocial behaviour (Miller & Eisenberg, 1998; de Wied *et al*., 2005; de Kemp *et al*., 2007; van Langen *et al*., 2014; van Hazebroek *et al*., 2017; Gantiva *et al*., 2021). Moreover, an early onset developmental trajectory has been identified, where early manifestations of aggression are followed by later delinquency, aggression, criminality, and in some cases, psychopathy (Farrington, 1989; Moffitt, 1993; Broidy *et al*., 2003; Jones *et al*., 2010; Dadds *et al*., 2012; Pingault *et al*., 2013; Robertson *et al*., 2018). Deficiencies in empathy are an important intra-individual risk factor for the development of such problematic behaviour and personality traits. Examining empathy in children is consequently key in order to identify its role and design interventions to disrupt potentially negative trajectories.

This line of investigation is particularly relevant in South Africa and other Low-Middle Income Countries (LMICs), where extremely high rates of violent crime and youth violence exist (Foster, 2012; de Ribera *et al*., 2019; Statistics SA, 2019). In South Africa, as in many other LMICs, macro societal factors including poverty and the legacy of colonialism are undoubtedly major contributors to this picture; however, understanding how individual factors such as empathy may contribute to these high rates remains an important consideration. Research has shown that interventions at the individual level can mitigate the effects of societal risk factors on individual vulnerabilities (van der Merwe & Dawes, 2007; Odgers *et al*., 2012). The ability to assess this fundamental social cognitive skill in development thus has broad applicability.

The precise definition and subsequent measurement of empathy remain contentious and variable. Many scholars consider empathy a multifaceted construct comprising at minimum both an affective and a cognitive component, where the affective component is concerned with emotion-sharing and the cognitive component with the ability to relate to others’ emotions or mental states on a cognitive level (Eisenberg & Fabes, 1990; Bischof-Köhler, 2012; Uribe *et al*., 2019). Thus, several measures of dispositional empathy have been designed informed by this theoretical conceptualisation.

Assessing dispositional or trait empathy is a cornerstone in many studies concerned with empathy, with self-report measures widely used in adolescent and adult samples. Measuring empathy in younger children remains a challenge, as they do not yet have the cognitive capacity to comment reliably on their own emotional and behavioural states. Consequently, it is common practice for parents to be asked to report on their child’s dispositional empathy (Dadds *et al*., 2008; Rieffe *et al*., 2010). While the extent to which parents can accurately reflect on their child’s empathic abilities is open to question, when coupled with other measures of child empathy and behaviour, parent-report measures can provide a valuable contribution to understanding child empathy and its development.

The Griffiths Empathy Measure (Dadds *et al*., 2008) is a well-known parent-report measure of dispositional cognitive and affective empathy which has previously demonstrated adequate reliability and validity in an Australian sample. While reasonable psychometric properties have been reported in a Chinese sample (Zhang. *et al*., 2014), Murphy (2019) did not find this to be the case in an analysis of the literature regarding this questionnaire. His analysis indicated a lack of construct validity for the scale. Murphy cites the lack of conceptual precision in the questions comprising the respective cognitive and affective subcomponents as the reason for poor performance. He argues that the cognitive subscale appears to be assessing primarily callousness, while the affective subscale seems to be assessing a specific aspect of affective empathy, namely emotion contagion. Despite the general agreement regarding the overarching separable aspects of cognitive and affective empathy, consensus at the finer-grained level regarding what specific subcomponents may comprise the construct continues to elude the field (see for e.g. Table 1 in the systematic review by de Lima & Osorio, 2021).

**Table 1.** Demographic information

	QCAE *n* = 440	QCAE-SGEM *n* = 280	SGEM *n* = 160	*t*/χ^2^	*p*	*d*/Cramer’s V
Child age (yrs)						
Range	4.58-13.92	4.58-13.92	6.08-13.92			
*M* (SD)	9,72(2,29)	10.02(2,30)	10.15(2.61)	0.54	0.588	0.05
Child Sex (M:F)	201:239	121:159	80:80	1.89	0.169	0.07
Child Ethnicity^a^	78:352:6:1	67:205:4:1	11:147:2:0			

*Note:* The QCAE sample includes the 160 primary caregivers who also completed the SGEM. Group contrasts were run using QCAE – SGEM and SGEM groups.

a Child Ethnicity: Black African: Coloured - Mixed Ancestry: Indian - South Asian: White; these terms include the South African terms as well as those recommended by the American Medical Association (Flanagin *et al*., 2021).

In a South African study using the Griffith Empathy Measure (GEM), Malcolm-Smith and colleagues (2015) demonstrated poor internal consistency reliability for the overall measure of dispositional empathy (Cronbach’s α = 0.47), poor discriminant validity and poor convergent validity. One potential explanation for this poor performance was that the response format (i.e., 9-point Likert scale) was too complex for parents to reliably report on their child’s dispositional empathy. This was evidenced in biased response patterns (i.e., only middle-of-the-range or extremes reported) along with interviewer reports of respondents’ difficulties with understanding the 9-point response format. The GEM thus did not appear to be an appropriate measure of child dispositional empathy in this South African LMIC context.

The Questionnaire of Cognitive and Affective Empathy (QCAE) (Reniers *et al*., 2011), another relatively recently developed measure assessing dispositional cognitive and affective empathy, has been broadly used in recent years. This self-report measure has been translated and yielded good psychometric results in a number of HICs, including Italy, Australia, France, and Portugal as well as in the original UK sample (Reniers *et al*., 2011; Myszkowski *et al*., 2017; Queiros *et al*., 2018; Di Girolamo *et al*., 2019; Gomez *et al*., 2022). The QCAE has also been used in a multisite study including HICs and LMICs, where (although its psychometric properties were not reported) similar associations between parent and child empathy, as well as age and child empathy, emerged across the various countries (Kozloff *et al*., 2021), indicating consistency in the QCAE’s performance across very different contexts. The QCAE’s broad applicability demonstrated across these contexts and China (an upper-middle income country; Liang *et al*., 2019) suggests it might prove appropriate for use in LMIC contexts.

Establishing a reliable and valid measure of empathy for the South African and other LMIC contexts is integral as poor measures undermine all findings. The aim of this study was therefore to examine the psychometric properties of the above-mentioned two-factor measures of child dispositional empathy in an LMIC, South African context. We assessed a version of the GEM using a simplified set of response options. We also set the question format of the QCAE to parent-report rather than self-report and assessed its performance. To our knowledge this is the first study to examine QCAE psychometric properties in this form. The goal was to determine whether either or both measures may be useful for assessing child empathy in a LMIC context.

## Methods

We used a cross-sectional design, with repeated measures, to assess the psychometric properties of two widely used measures of dispositional cognitive and affective empathy. We examined a simplified 3-point response format version of the GEM (Simplified GEM or SGEM), along with that of the QCAE being used in a larger ongoing protocol. All analyses were conducted using R Studio (2020). SGEM data (*n* = 160) was compared to that of the QCAE (*n* = 440; including the 160 who completed the SGEM). We assessed internal consistency reliability using Cronbach’s alpha, and correlated cognitive and affective subscale scores from participants who completed both questionnaires to assess convergent validity. Subsequently, given poor performance of the SGEM, Confirmatory Factor Analysis was utilised to investigate the integrity of the two-factor structure (i.e., cognitive and affective subscales), as well as the 5-factor structure proposed by the authors, of the QCAE only. This analysis also yielded composite reliability scores for the QCAE.

### Participants

The study adhered to principles for research with human participants outlined in the Declaration of Helsinki (World Medical Association, 2013) and ethical approval was granted by the Ethics Committee of the University of Cape Town’s Department of Psychology (PSY2013-001). Participants were recruited from several English-medium public schools serving low-mid SES communities in Cape Town. Recruitment proceeded with permission from the Western Cape Education Department, and all parents or primary caregivers provided written informed consent. Parents/primary caregivers self-identified as belonging to South African ethnic categories that remain in consistent use due to post-Apartheid attempts to achieve employment equity and ensure diversity in educational and other institutions. The majority of the sample (80%) identified as ‘Coloured’, a mixed ancestry group who have a unique identity, and form a large part of the Western Cape population; around 18% identified as Black African; with very small numbers of Indian (South Asian, 1%) and White (0.02%) participants. The sample is thus representative of the province’s population (Statistics South Africa, Census 2019; Provincial Profile Western Cape, 2004).

As part of a larger ongoing study protocol, primary caregivers were asked to complete several parent-report questionnaires including the QCAE. Due to concerns about the GEM outlined above, it was not initially used in this protocol, and we only introduced the SGEM later to a portion of the ongoing study participants who had yet to provide data, in order to assess its psychometric performance. Consequently, a total of 160 primary caregivers completed the SGEM while 440 completed the QCAE. Although different, these sample sizes were nonetheless sufficient for the required data analyses (Bujang *et al*., 2018). Furthermore, all of those who completed the SGEM also completed the QCAE. Demographic information of the sample is presented in Table 1.

### Materials

A simplified version of the GEM (SGEM; Dadds *et al*., 2008) was used as one of two measures assessing dispositional empathy (i.e., cognitive and affective trait empathy).

T. E. Moffitt (personal communication, February 2014) suggested that a simplified response scale might be required to maximise parent comprehension and hence enable reliable reporting; based on her extensive experience with cohort studies, parents frequently do not understand the fine gradations present in complex response formats. To reduce its complexity, the original 9-point Likert scale format was thus changed to a 3-point Likert scale format with permission from the developers. In the original GEM, response options ranged from −4 to 4, with end-point anchors only (i.e., *strongly disagree* = −4 and *strongly agree* = 4), while the response options for the SGEM were *never* (1), *sometimes* (2) or *always* (3). This scale consists of 23 items of which 14 assess cognitive empathy and 9 assess affective empathy. Primary caregivers reported on their child’s empathy.

The Questionnaire of Cognitive and Affective Empathy (QCAE) developed by Reniers *et al.* (2011) also measures dispositional cognitive and affective empathy. This scale consists of 32 items of which 17 assess cognitive empathy and 15 assess affective empathy. Responses are made on a 4-point Likert scale with four anchored response options: *strongly disagree* (1), *disagree* (2), *agree* (3), *strongly agree* (4). This scale was developed drawing from several widely used and validated questionnaires measuring empathy (see Reniers *et al*., 2011). Once again, primary caregivers reported on their child’s empathy.

## Results

### Reliability analyses

Internal consistency was assessed for each of the questionnaires (i.e., the overall scale) as well as the affective and cognitive subscales by means of Cronbach’s alpha coefficient. Values are presented in Table 2 below. Composite reliability for the QCAE was calculated via the CFA, and values are presented in that section below. As can be seen, the SGEM performed poorly, with consistently low alpha values. In contrast, the QCAE performed well. As is generally seen in the literature, alpha values were excellent for the overall scale and the cognitive subscale but only satisfactory for the affective subscale.

**Table 2.** Internal consistency reliability for the SGEM and QCAE

	*n*	Overall scale	Cognitive subscale	Affective subscale
SGEM	160	0.63	0.48	0.64
QCAE	440	0.89	0.90	0.72

(Note that The data and code used in this report are available at https://osf.io/56x73/?view_only=de2327a53fe44ef8b2f2ee6794371b37)

Despite performing considerably better than the original version utilised in the Malcolm-Smith *et al*. (2015) article (α = 0.47), the SGEM’s performance was less than satisfactory, overall α = .63. Both subscales also performed poorly. Inspection of the inter-item correlation matrix showed low correlations and multiple negative correlations (See supplementary material, Table 1). Furthermore, item-total correlations revealed that removing items would not make a significant difference to the internal consistency of the scale (supplementary material, Table 2).

The QCAE performed well, with an overall scale alpha of 0.89. Despite this, there were still some low and negative inter-item correlations. Item 1 specifically, was flagged as problematic, with a negative corrected item-total correlation. Furthermore, the affective subscale alpha of 0.72, while satisfactory, is less than ideal. However, removing 4 items would improve this alpha only slightly, to .74.

### Convergence between the measures

Given the poor reliability of the SGEM, it is perhaps not surprising that the SGEM and QCAE scores did not converge. Specifically, scores for those 160 participants who completed both the SGEM and the QCAE were not correlated: overall scale: *r* = −0.01, *p* = .885; cognitive subscale: *r* = −0.04, *p* = .610; affective subscale: *r* = 0.03, *p* = .740.

### Confirmatory Factor Analysis (CFA) of the QCAE

Because of the poor internal consistency values for the SGEM and poor convergence between the SGEM and the QCAE, no further analysis was conducted for the SGEM. CFA was employed to confirm the structural validity of the QCAE. CFA analyses were conducted using the R-package ‘lavaan’ (Rosseel, 2012). Initial screening of the data indicated that the QCAE responses did not follow a normal distribution (Mardia multivariate kurtosis =47.43, *p* < 0.001). To account for this, maximum likelihood estimation with robust standard errors was used to compute the goodness of fit indices of the models (Lai, 2018). An acceptable fit would be indicated by CFI > 0.90 and RMSEA ≤ 0.06 (Hu & Bentler, 1999). If the resulting model did not have an acceptable fit, items whose factor loadings were <0.4 were removed (Stevens, 1992). Furthermore, residual errors and modification indices were examined to identify covariance among items and those that fitted theoretically were included to improve the overall fit of the model (McDonald & Ho, 2002).

A two-factor CFA model tested the item loadings of the proposed Cognitive and Affective subscales of the QCAE. Goodness of fit measures indicated that the initial model did not fit the data well, with CFI = 0.76 and RMSEA = 0.08 (90% CI: 0.07–0.08). Inspection of the factor loadings flagged items 1, 2, 17, 23, and 29 as problematic as their loadings were well below the acceptable value of 0.4 (factor loadings = 0.21, 0.01, −0.17, 0.24, −0.09 respectively; supplementary material, Table 3). After removing these five items from the analysis the two-factor model continued to fit the data poorly (CFI = 0.82 and RMSEA = 0.08 [90% CI: 0.07–0.08]). Residual errors and modification indices revealed that the poor fit was likely due to covariation between, (a) items 30 and 31, (b) items 3 and 4, (c) items 5 and 6, (d) items 25 and 27, and (e) items 16 and 26. Careful examination of the QCAE revealed that these items assess similar aspects of cognitive and affective empathy, indicating some redundancy in items. A two-factor model accounting for these inter-item correlations fit the data well, with CFI = 0.90 and RMSEA = 0.058 (90% CI: 0.05–0.06; see Table 3). Composite reliability for this model was very good: overall scale = 0.94; cognitive subscale = 0.92; affective subscale = 0.80.

**Table 3.** Confirmatory factor analysis results for the QCAE – 2-factor model

Factor	Item	Standardised factor loadings	Standard error	95% Confidence intervals
Cognitive empathy	3	0.46*	0.05	[0.37, 0.55]
	4	0.56*	0.04	[0.48, 0.64]
	5	0.55*	0.04	[0.48, 0.63]
	6	0.58*	0.04	[0.50, 0.65]
	15	0.59*	0.04	[0.51, 0.67]
	16	0.62*	0.04	[0.55, 0.70]
	18	0.64*	0.03	[0.58, 0.70]
	19	0.64*	0.04	[0.57, 0.71]
	20	0.72*	0.03	[0.67, 0.77]
	21	0.64*	0.04	[0.57, 0.71]
	22	0.63*	0.04	[0.56, 0.71]
	24	0.64*	0.03	[0.57, 0.71]
	25	0.70*	0.03	[0.64, 0.75]
	26	0.69*	0.03	[0.63, 0.76]
	27	0.62*	0.04	[0.54, 0.69]
	28	0.59*	0.04	[0.52, 0.66]
	30	0.61*	0.04	[0.53, 0.68]
	31	0.55*	0.04	[0.46, 0.63]
Affective empathy	7	0.48*	0.05	[0.39, 0.58]
	8	0.60*	0.05	[0.51, 0.69]
	9	0.52*	0.05	[0.43, 0.61]
	10	0.65*	0.04	[0.57, 0.73]
	11	0.46*	0.05	[0.36, 0.56]
	12	0.57*	0.04	[0.49, 0.66]
	13	0.59*	0.05	[0.50, 0.68]
	14	0.71*	0.03	[0.65, 0.78]

*p < 0.0001.

Next, we performed a CFA using the five-factor structure suggested by the original authors (Reniers *et al*., 2011). We found that items in the Peripheral Responsivity subscale (items 2, 11, 17, 19), as well as items 1 and 23 did not demonstrate acceptable factor loadings and were removed from the model (supplementary material, Table 4). Additionally, covariation between items 30 and 31 and items 3 and 4 were accounted for to achieve a satisfactory model fit. The resulting four-factor model appears to be the best fit for the data (CFI = 0.91, RMSEA = 0.06 [90% CI: 0.05–0.06], see Table 4). The composite reliability scores of the factors within the four-factor structure are relatively good (perspective taking = 0.89; online simulation = 0.85; emotion contagion = 0.73, and proximal responsivity = 0.61). The total composite reliability for this four-factor structure was 0.95.

**Table 4.** Confirmatory factor analysis results for the QCAE – 4-factor model

Factor	Item	Standardised factor loadings	Standard error	95% Confidence intervals
Perspective Taking	15	0.59*	0.04	[0.51, 0.67]
	16	0.69*	0.03	[0.62, 0.75]
	19	0.63*	0.04	[0.56, 0.71]
	20	0.70*	0.03	[0.65, 0.76]
	21	0.60*	0.04	[0.52, 0.68]
	22	0.66*	0.03	[0.52, 0.68]
	24	0.65*	0.03	[0.58, 0.71]
	25	0.75*	0.02	[0.70, 0.80]
	26	0.76*	0.03	[0.71, 0.81]
	27	0.68*	0.03	[0.62, 0.75]
Online simulation	3	0.51*	0.05	[0.43, 0.60]
	4	0.65*	0.04	[0.58, 0.72]
	5	0.69*	0.03	[0.63, 0.76]
	6	0.70*	0.03	[0.64, 0.76]
	18	0.66*	0.03	[0.59, 0.73]
	28	0.60*	0.04	[0.53, 0.68]
	30	0.68*	0.03	[0.61, 0.74]
	31	0.61*	0.04	[0.53, 0.69]
Emotion contagion	8	0.64*	0.04	[0.55, 0.73]
	9	0.58*	0.05	[0.48, 0.67]
	13	0.60*	0.05	[0.51, 0.69]
	14	0.71*	0.04	[0.64, 0.79]
Proximal responsivity	7	0.51*	0.05	[0.41, 0.61]
	10	0.66*	0.05	[0.56, 0.76]
	12	0.57*	0.05	[0.48, 0.66]

*p < 0.0001.

## Discussion

Our results indicate that the QCAE may be a useful parent-report measure of dispositional empathy for school-aged children in South Africa and other LMIC contexts. As the first examination of a parent-report version of the questionnaire these results are very promising: The scale evidenced good reliability, and confirmatory factor analysis indicated that, once item covariance was accounted for, the two-factor structure most commonly examined in empathy research (viz. cognitive and affective empathy subscales) fitted our data appropriately. A four-factor model, consistent with those reported by the original authors but excluding the peripheral responsivity subscale, fit our data well.

The SGEM, in contrast, did not perform optimally. Our initial concern regarding the GEM was that the complex response format might have been the cause of unreliable reporting in the 2015 Malcolm-Smith *et al.*, study. The 9-point Likert scale features only end-point anchors (strongly disagree and strongly agree), and although fine-grained scaling of responses is often considered ideal, it is likely that parents in LMIC contexts would not be able to make meaningful sense of the many available but unlabelled scale points. We therefore simplified the response format to three easily understandable options - *never*, *sometimes,* or *always.* However, even with this simplified response format, the internal consistency reliability of this measure, although improved, was not satisfactory (see Table 2).

The fact that scores from the subsample that completed both the SGEM and the QCAE, two measures ostensibly assessing the same construct, and both featuring cognitive and affective subscales, did not correlate at all (all *r* values < 0.05; all *p* values > 0.60) raises serious questions about the validity of the SGEM. Our findings concur with those of Murphy (2019), who also raises serious concerns about the scale’s validity. Moreover, as an unreliable instrument cannot be valid, we investigated construct validity via confirmatory factor analysis only in the QCAE.

The QCAE evidenced good reliability, both in terms of Cronbach’s alpha (see Table 2) and composite reliability values. We then examined whether the factor structure proposed by the original authors (Reniers *et al*., 2011) was evident in our data. Confirmatory factor analysis indicated that once covariance between a number of items was allowed, the two-factor model for cognitive and affective empathy subscales attained acceptable fit. Studies in Italy and China (Di Girolamo *et al*., 2019; Liang *et al*., 2019) also found that the two-factor model fit their data, although researchers in France found that the 5-factor model fit their data better than a 2-factor model (Myszkowski *et al*., 2017). As the subdomains of cognitive and affective empathy are generally of central interest to researchers, it was important to confirm that these constructs are adequately captured by the questionnaire in our context.

We also attempted to confirm the more complex 5-factor structure seen by Reniers and colleagues (2011). Our data, however, did not fit this model, and CFA indicated that a four-factor structure was instead supported. These four factors are consistent with four of the five reported by Reniers et al., However, the peripheral responsivity subscale factor did not emerge. The original authors also found this problem, and applied corrections to improve their model. Queiros *et al* (2018) also noted difficulties with the peripheral responsivity subscale and point to studies with clinical samples (Michaels *et al*., 2014; Horan *et al*., 2015) where its reliability and convergent validity were problematic. Similarly, Liang *et al*. (2019) found poor performance for the peripheral responsivity subscale, and in a meta-analysis, de Lima and Osorio (2021) found low reliability for the peripheral responsivity subscale. Authors who wish to examine the five QCAE subscales should be cautious regarding interpretation of the peripheral responsivity subscale.

Other very recent studies term the factor structure of the QCAE undecided (de Lima & Osorio, 2021; Gomez *et al*., 2022). Gomez *et al*. (2022) critique the use of parcelling in factor analyses in several psychometric studies of the QCAE (Reniers *et al*., 2011; Horan *et al*., 2015; Myszkowski *et al*., 2017; Queiros *et al*., 2018; Di Girolamo *et al*., 2019; Liang *et al*., 2019), arguing that this method is only appropriate where there is clear evidence for the factor structure at the item level, and thus calling these studies’ results into question. In line with the recommendation from Gomez et al., our analysis was conducted at the item-level. The recent meta-analysis of empathy measures also points to difficulties regarding factor structure in various instruments (de Lima & Osorio, 2021). As these authors argue, it is entirely likely that differences in the conceptualisation of the precise subcomponents of empathy impact on scale construction, and thus contribute to measurement difficulties. They concur that the agreement around the broader but differentiable components of cognitive and affective empathy is stronger. At present it is thus probably preferable to assess empathy in terms of these broader aspects or subscales.

Our results indicate that we may have some confidence in results generated via use of the parent-report QCAE in the South African context. These results also indicate that the QCAE can be used not only as a self-report measure as in previous studies, but also as a parent-report measure – an important tool when studying empathy development. The question of how accurately parents are able to report on their child’s empathic disposition remains a concern, but the evidence presented here indicates that data from the parent-report version of the QCAE, ideally in combination with other more direct measures of empathy, can yield valuable information.

This questionnaire may also be useful in other LMIC contexts, where similar factors pertain, such as low SES, low levels and poor quality of education, as well as multiple languages in the population. In such contexts, measures developed and found suitable for HICs are often applied, but their suitability and psychometric properties are seldom thoroughly interrogated (Malcolm-Smith *et al*., 2023; Zieff *et al*., 2023).

Our study features several limitations we must acknowledge. Although sufficient for the analyses conducted, our sample was relatively small, and included only parents in the Cape Town metropolitan region in the Western Cape of South Africa. Investigation of this measure in other regions, particularly more rural areas, would strengthen confidence in its broad applicability. We would ideally have liked to collect more SGEM responses, but the late stage of the ongoing study including the QCAE prohibited this. The SGEM sample size was sufficient for the reliability analysis, and correlation analyses between the two measures were only conducted for participants who had completed both questionnaires. It would of course be desirable to assess convergent validity for the QCAE with other empathy measures, however as no validated scale assessing empathy exists for South Africa, we were unfortunately unable to do this.

We assessed these parent-report questionnaires for school-aged children only. Future studies should include assessment of the self-report version of the QCAE for older children and adults in South African or other LMIC contexts.

Regarding the QCAE, item 1 seems to be particularly problematic. We note that the covariance between a number of QCAE items indicates some redundancy in the questionnaire, and a few items did not load particularly well in the two-factor structure. Researchers using the QCAE are thus faced with a choice between omitting certain items, or proceeding with caution as the removal of items from standard questionnaires makes comparisons across studies problematic. In the absence of a standard revised version of the questionnaire, our general preference would be to retain cross study comparability by using standard question sets, while acknowledging that most such questionnaires are relatively blunt instruments.

## Conclusion

This investigation of two well-known questionnaire measures of dispositional empathy in a LMIC context provides evidence that the parent-report version of the QCAE is an appropriate scale for use in school-aged children in such contexts. The ability to assess empathy is critical, particularly in young children, where interventions and social skills training can have long-term individual and societal benefits (van der Merwe & Dawes, 2007; Malti *et al*., 2016; Spinrad & Gal, 2018). Researchers working in LMIC contexts are encouraged to thoroughly examine the psychometric properties of well-used instruments developed in westernised HIC contexts, as the assumption that they will perform similarly in very different populations may not be correct.

## Supporting information

Malcolm-Smith et al. supplementary material Malcolm-Smith et al. supplementary material

## Supplementary material

The supplementary material for this article can be found at https://doi.org/10.1017/neu.2024.19.
