# Supplementary material for: Measuring dispositional empathy in South African children
Source: Acta Neuropsychiatr. 2024 Apr 25;37:e26. doi: 10.1017/neu.2024.19 (PMC13130298; doi:10.1017/neu.2024.19)
Supplement: Malcolm-Smith et al. supplementary material [file S092427082400019Xsup001.docx]

**Supplementary Material**

**Table 1**

*Inter-Item Correlation Matrix for SGEM*

| Item | 1 | 2 | 3 | 4 | 5 | 6 | 7 | 8 | 9 | 10 | 11 | 12 | 13 | 14 | 15 | 16 | 17 | 18 | 19 | 20 | 21 | 22 | 23 |
| --- | --- | --- | --- | --- | --- | --- | --- | --- | --- | --- | --- | --- | --- | --- | --- | --- | --- | --- | --- | --- | --- | --- | --- |
| 1 | - | .18 | -.12 | .43 | .34 | -.14 | .13 | .17 | .20 | .17 | .26 | .41 | .04 | .05 | .15 | .07 | -.01 | .36 | .28 | .01 | .04 | .17 | .04 |
| 2 |  | - | -.01 | .19 | .15 | -.12 | .08 | .11 | .01 | .12 | .06 | .03 | .02 | .14 | .10 | .03 | .13 | .47 | .12 | -.05 | .05 | -.06 | -.11 |
| 3 |  |  | - | -.20 | -.24 | .14 | -.20 | -.12 | -.15 | -.01 | -.25 | -.21 | .11 | -.02 | -.36 | -.26 | .16 | -.07 | -.21 | .20 | -.04 | -.06 | -.17 |
| 4 |  |  |  | - | .40 | -.08 | .18 | .04 | .13 | .12 | .33 | .29 | .04 | .17 | .20 | .18 | -.09 | .25 | .09 | -.10 | .15 | .17 | .04 |
| 5 |  |  |  |  | - | -.07 | .13 | .23 | .27 | -.02 | .38 | .25 | .01 | .11 | .20 | .26 | -.20 | .19 | .27 | -.03 | .03 | .11 | .22 |
| 6 |  |  |  |  |  | - | -.17 | .01 | -.09 | -.04 | -.18 | -.14 | .03 | .03 | -.13 | -.15 | .18 | -.04 | -.14 | .03 | -.09 | .07 | -.08 |
| 7 |  |  |  |  |  |  | - | .12 | .10 | .10 | .27 | .20 | -.09 | .24 | .34 | .26 | -.15 | .11 | .09 | -.15 | .09 | .09 | .01 |
| 8 |  |  |  |  |  |  |  | - | .37 | -.06 | .17 | .12 | -.09 | .16 | .10 | .22 | -.02 | .07 | .14 | -.13 | -.01 | .08 | .11 |
| 9 |  |  |  |  |  |  |  |  | - | -.25 | .28 | .13 | .17 | .13 | .06 | .35 | -.12 | -.02 | .20 | -.02 | -.06 | .07 | .13 |
| 10 |  |  |  |  |  |  |  |  |  | - | .01 | .18 | .08 | .02 | .04 | -.01 | .03 | .25 | .15 | -.04 | .10 | -.03 | -.14 |
| 11 |  |  |  |  |  |  |  |  |  |  | - | .42 | -.07 | .28 | .29 | .49 | -.22 | .16 | .17 | -.12 | .10 | .23 | .22 |
| 12 |  |  |  |  |  |  |  |  |  |  |  | - | .05 | .20 | .27 | .20 | -.02 | .33 | .36 | .02 | .18 | .15 | -.01 |
| 13 |  |  |  |  |  |  |  |  |  |  |  |  | - | -.01 | -.10 | -.09 | .29 | .01 | .08 | .39 | .15 | -.05 | .09 |
| 14 |  |  |  |  |  |  |  |  |  |  |  |  |  | - | .12 | .24 | -.04 | .16 | .17 | -.06 | .09 | .22 | .04 |
| 15 |  |  |  |  |  |  |  |  |  |  |  |  |  |  | - | .24 | -.03 | .16 | .12 | -.16 | .20 | .17 | -.01 |
| 16 |  |  |  |  |  |  |  |  |  |  |  |  |  |  |  | - | -.20 | .14 | .15 | -.21 | -.06 | .12 | .25 |
| 17 |  |  |  |  |  |  |  |  |  |  |  |  |  |  |  |  | - | .01 | .04 | .30 | .04 | -.01 | .03 |
| 18 |  |  |  |  |  |  |  |  |  |  |  |  |  |  |  |  |  | - | .18 | .02 | .04 | .09 | -.08 |
| 19 |  |  |  |  |  |  |  |  |  |  |  |  |  |  |  |  |  |  | - | .01 | .12 | .18 | .11 |
| 20 |  |  |  |  |  |  |  |  |  |  |  |  |  |  |  |  |  |  |  | - | .18 | -.05 | .09 |
| 21 |  |  |  |  |  |  |  |  |  |  |  |  |  |  |  |  |  |  |  |  | - | .01 | .08 |
| 22 |  |  |  |  |  |  |  |  |  |  |  |  |  |  |  |  |  |  |  |  |  | - | -.07 |
| 23 |  |  |  |  |  |  |  |  |  |  |  |  |  |  |  |  |  |  |  |  |  |  | - |

**Table 2**

*Item-Total Statistics for the SGEM*

| Item | Corrected Item-Total Correlation | Cronbach's Alpha if Item Deleted |
| --- | --- | --- |
| 1 | .43 | .60 |
| 2 | .21 | .62 |
| 3 | -.27 | .68 |
| 4 | .40 | .60 |
| 5 | .40 | .60 |
| 6 | -.15 | .66 |
| 7 | .24 | .62 |
| 8 | .24 | .62 |
| 9 | .25 | .62 |
| 10 | .10 | .64 |
| 11 | .45 | .59 |
| 12 | .47 | .59 |
| 13 | .12 | .63 |
| 14 | .34 | .61 |
| 15 | .26 | .61 |
| 16 | .31 | .61 |
| 17 | .01 | .64 |
| 18 | .38 | .60 |
| 19 | .36 | .60 |
| 20 | .03 | .63 |
| 21 | .18 | .62 |
| 22 | .20 | .62 |
| 23 | .10 | .63 |

***Table 3***

*Original Confirmatory Factor Analysis Results for the QCAE – 2-Factor Model*

| Factor | Item | Standardized Factor Loadings | Standard Error | 95%  Confidence Intervals |
| --- | --- | --- | --- | --- |
| Cognitive Empathy |  |  |  |  |
|  | 1 | 0.21 | 0.05 | (0.10, 0.31) |
|  | 3 | 0.49* | 0.04 | (0.40, 0.58) |
|  | 4 | 0.58* | 0.04 | (0.50, 0.66) |
|  | 5 | 0.58* | 0.04 | (0.51, 0.66) |
|  | 6 | 0.60* | 0.04 | (0.53, 0.68) |
|  | 15 | 0.58* | 0.04 | (0.51, 0.66) |
|  | 16 | 0.63* | 0.04 | (0.55, 0.70) |
|  | 18 | 0.64* | 0.03 | [0.57-0.70] |
|  | 19 | 0.63* | 0.04 | (0.56, 0.70) |
|  | 20 | 0.70* | 0.03 | (0.65, 0.76) |
|  | 21 | 0.63* | 0.04 | (0.56, 0.70) |
|  | 22 | 0.62* | 0.04 | (0.54, 0.69) |
|  | 24 | 0.64* | 0.03 | (0.57, 0.70) |
|  | 25 | 0.70* | 0.03 | (0.65, 0.76) |
|  | 26 | 0.70* | 0.03 | (0.64, 0.76) |
|  | 27 | 0.63* | 0.04 | (0.55, 0.70) |
|  | 28 | 0.60* | 0.03 | [0.53, 0.67] |
|  | 30 | 0.63* | 0.04 | [0.56, 0.70] |
|  | 31 | 0.58* | 0.04 | [0.49, 0.66] |
| Affective Empathy |  |  |  |  |
|  | 2 | 0.00 | 0.06 | [-0.12, 0.12] |
|  | 7 | 0.50* | 0.05 | 0.41, 0.59] |
|  | 8 | 0.58* | 0.05 | [0.49, 0.68] |
|  | 9 | 0.51* | 0.05 | [0.41, 0.60] |
|  | 10 | 0.65* | 0.04 | [0.57, 0.73] |
|  | 11 | 0.47* | 0.05 | [0.37, 0.57] |
|  | 12 | 0.58* | 0.04 | [0.50, 0.66] |
|  | 13 | 0.58* | 0.05 | [0.48, 0.67] |
|  | 14 | 0.71* | 0.03 | [0.64, 0.78] |
|  | 17 | -0.17 | 0.06 | [-0.28, -0.06] |
|  | 23 | 0.24 | 0.06 | [0.11, 0.36] |
|  | 29 | -0.09 | 0.06 | [-0.21, 0.02] |

**p*<0.0001

Table 4

*Confirmatory Factor Analysis Results for the QCAE – 5-Factor Model*

| Factor | Item | Standardized Factor Loadings | Standard Error | 95%  Confidence Intervals |
| --- | --- | --- | --- | --- |
| Perspective Taking |  |  |  |  |
|  | 15 | 0.59* | 0.04 | [0.51;0.67] |
|  | 16 | 0.68* | 0.03 | [0.62;0.75] |
|  | 19 | 0.63* | 0.04 | [0.56;0.71] |
|  | 20 | 0.7* | 0.03 | [0.65;0.76] |
|  | 21 | 0.6* | 0.04 | [0.52;0.68] |
|  | 22 | 0.66* | 0.03 | [0.6;0.73] |
|  | 24 | 0.65* | 0.03 | [0.58;0.71] |
|  | 25 | 0.75* | 0.02 | [0.7;0.8] |
|  | 26 | 0.76* | 0.03 | [0.71;0.81] |
|  | 27 | 0.68* | 0.03 | [0.62;0.75] |
| Online Simulation |  |  |  |  |
|  | 1 | 0.23* | 0.05 | [0.13;0.33] |
|  | 3 | 0.54* | 0.05 | [0.45;0.63] |
|  | 4 | 0.66* | 0.04 | [0.59;0.73] |
|  | 5 | 0.68* | 0.03 | [0.61;0.74] |
|  | 6 | 0.69* | 0.03 | [0.63;0.76] |
|  | 18 | 0.65* | 0.04 | [0.58;0.72] |
|  | 28 | 0.6* | 0.04 | [0.53;0.67] |
|  | 30 | 0.72* | 0.03 | [0.66;0.79] |
|  | 31 | 0.67* | 0.04 | [0.59;0.75] |
| Emotion Contagion |  |  |  |  |
|  | 8 | 0.63* | 0.05 | [0.55;0.72] |
|  | 9 | 0.57* | 0.05 | [0.48;0.67] |
|  | 13 | 0.59* | 0.05 | [0.5;0.69] |
|  | 14 | 0.73* | 0.04 | [0.65;0.8] |
| Proximal Responsivity |  |  |  |  |
|  | 7 | 0.52* | 0.05 | [0.43;0.61] |
|  | 10 | 0.63* | 0.05 | [0.53;0.74] |
|  | 12 | 0.56* | 0.05 | [0.46;0.65] |
|  | 23 | 0.36* | 0.07 | [0.23;0.5] |
| Peripheral Responsivity |  |  |  |  |
|  | 2 | 0.41 | 0.21 | [0;0.82] |
|  | 11 | -0.14 | 0.07 | [-0.28;-0.01] |
|  | 17 | 0.14 | 0.15 | [-0.15;0.43] |
|  | 29 | 0.7 | 0.37 | [-0.03;1.44] |
